# N, LNR or LODDS: Which Is the Most Appropriate Lymph Node Classification Scheme for Patients with Radically Resected Pancreatic Cancer?

**DOI:** 10.3390/cancers14071834

**Published:** 2022-04-06

**Authors:** Dimitrios Prassas, Sami Alexander Safi, Maria Chara Stylianidi, Leila Anne Telan, Sarah Krieg, Christoph Roderburg, Irene Esposito, Tom Luedde, Wolfram Trudo Knoefel, Andreas Krieg

**Affiliations:** 1 Department of Surgery (A), Heinrich-Heine-University and University Hospital Duesseldorf, Moorenstr. 5, 40225 Duesseldorf, Germany; dimitrios.prassas@med.uni-duesseldorf.de (D.P.); sami-alexander.safi@med.uni-duesseldorf.de (S.A.S.); maria-chara.stylianidi@med.uni-duesseldorf.de (M.C.S.); leilaanne.telan@med.uni-duesseldorf.de (L.A.T.); 2 Clinic for Gastroenterology, Hepatology and Infectious Diseases, Heinrich-Heine-University and University Hospital Duesseldorf, Moorenstr. 5, 40225 Duesseldorf, Germany; sarah.krieg@med.uni-duesseldorf.de (S.K.); christoph.roderburg@med.uni-duesseldorf.de (C.R.); tom.luedde@med.uni-duesseldorf.de (T.L.); 3 Institute of Pathology, Heinrich-Heine-University and University Hospital Duesseldorf, Moorenstr. 5, 40225 Duesseldorf, Germany; irene.esposito@med.uni-duesseldorf.de

**Keywords:** LODDS, LNR, lymph node classification, pancreatic cancer

## Abstract

**Simple Summary:**

To date, no data are available regarding the most appropriate alternative LN classification system with respect to prognostic power and discriminative ability in cases with resectable pancreatic ductal adenocarcinoma (PDAC). We compared different lymph node classification systems with regard to accurate evaluation of overall survival in 319 patients with resected PDAC. One LNR and one LODDS classification scheme were found to out-perform the N category in distinct patient subgroups. Only the LODDS classification exhibited statistically significant, gradually increasing HRs of their subcategories and, at the same time, significantly better discriminative potential in the subgroups of patients with PDAC of the head or corpus and in patients with tumor-free resection margins or M0 status, respectively.

**Abstract:**

Background: Even though numerous novel lymph node (LN) classification schemes exist, an extensive comparison of their performance in patients with resected pancreatic ductal adenocarcinoma (PDAC) has not yet been performed. Method: We investigated the prognostic performance and discriminative ability of 25 different LN ratio (LNR) and 27 log odds of metastatic LN (LODDS) classifications by means of Cox regression and C-statistic in 319 patients with resected PDAC. Regression models were adjusted for age, sex, T category, grading, localization, presence of metastatic disease, positivity of resection margins, and neoadjuvant therapy. Results: Both LNR or LODDS as continuous variables were associated with advanced tumor stage, distant metastasis, positive resection margins, and PDAC of the head or corpus. Two distinct LN classifications, one LODDS and one LNR, were found to be superior to the N category in the complete patient collective. However, only the LODDS classification exhibited statistically significant, gradually increasing HRs of their subcategories and at the same time significantly higher discriminative potential in the subgroups of patients with PDAC of the head or corpus and in patients with tumor free resection margins or M0 status, respectively. On this basis, we built a clinically helpful nomogram to estimate the prognosis of patients after radically resected PDAC. Conclusion: One LNR and one LODDS classification scheme were found to out-perform the N category in terms of both prognostic performance and discriminative ability, in distinct patient subgroups, with reference to OS in patients with resected PDAC.

## 1. Introduction

Pancreatic adenocarcinoma (PDAC) remains a highly lethal malignancy with a poor 5-year overall survival (OS) rate of 8–14% in the western world [1]. Although it has been ranked as the 14th most common solid malignant tumor in Europe in 2019, it is the 4th cause of cancer-related death, which reflects its remarkable biological aggressiveness [2]. Perturbingly, long-term estimations reveal rising trends of incidence and mortality, which necessitates further advances in the prevention and treatment of the disease1. Surgical resection constitutes the therapeutic cornerstone in PDAC. Among factors that interact with the postoperative oncologic outcome such as chemotherapy and tumor size, lymph node (LN) status also plays a decisive role as a predictor of survival [3,4]. Currently, the Tumor Node Metastasis (TNM) classification by the American Joint Committee on Cancer (AJCC) and the Union for International Cancer Control (UICC) is the most commonly used classification system worldwide [5,6]. However, this classification takes into account the nodal positivity but not the extent of total nodal yield. In the last decade, alternative lymph node classification schemes such as the lymph node ratio (LNR) and log odds of positive lymph nodes (LODDS) have been introduced as alternatives to the classic TNM classification. Existing data provide evidence on the suitability of LODDS as a predictor for OS in various cancer entities including PDAC [7,8]. Nevertheless, a classification scheme is clinically appropriate when utilized as a categorical variable, with existing distinct subcategories. These subcategories vary as proposed cut-off values of LNR and LODDS classifications and are also remarkably diverse. To date, no data are available regarding the most appropriate alternative LN classification system with respect to prognostic power and discriminative ability in cases with resectable PDAC. The aim of the present work was to investigate the above-mentioned issue in patients with pancreatic cancer who underwent surgical resection in our department and to identify the most appropriate sets of cut-off values for alternative LN classifications.

## 2. Materials and Methods

### 2.1. Patients and Procedures

The present study includes prospectively collected data from 319 patients with PDAC who underwent radical surgery with curative intent at the University Hospital Duesseldorf between 2003 and 2020. Demographic data as well as operative and clinicopathological data were analyzed from the prospectively maintained database of our department. Patients with incomplete histopathological information, death within 30 days postoperatively, and patients lost to follow up were excluded. Only cases with resectable or borderline resectable tumors were enrolled. All surgeries were performed by experienced pancreatic surgeons. Patients underwent at least a standard lymphadenectomy. An extended lymphadenectomy was also carried out in most of the cases, as described in the consensus conference of the international study group on pancreatic surgery (ISGPS) held in 2013 to unify the terminology [9]. More specifically, removal of regional LNs (standard regional lymphadenectomy) was followed by the skeletonization of the hepatic arteries (LN station 8), superior mesenteric artery (LN station 14), between aorta and inferior pancreaticoduodenal artery, celiac trunk (LN station 9), and dissection of the anterolateral aspect of the aorta and vena cava. All cases were discussed both pre- and postoperatively in our multidisciplinary tumor board. Neoadjuvant chemotherapy was reserved for cases with anatomically borderline resectable tumors, in line with the International Association of Pancreatology/European Pancreatic Club guidelines for the treatment of pancreatic cancer [10]. Anatomical borderline resectability was defined as tumor contact with the superior mesenteric artery and/or celiac artery of less than 180° without signs of stenosis or deformity, tumor contact with the common hepatic artery without signs of tumor contact with the proper hepatic artery and/or celiac artery, and tumor contact with the superior mesenteric vein and/or portal vein including bilateral narrowing or occlusion without extending beyond the inferior border of the duodenum, as originally proposed by the work of Callery et al. [11]. Patients were routinely followed every three to six months until their death. Overall survival (OS) was defined as the time span between surgery and death from any cause and was used as the primary endpoint. 

The study was carried out in accordance with the principles of good clinical practice and the Declaration of Helsinki. Informed consent was not possible because the vast majority of the included patients are deceased. All data were anonymized from the source, and there is no evidence that the patients would have objected to their collection and analysis. An institutional review board (IRB)-approval of the Medical Faculty, Heinrich-Heine-University Duesseldorf was retrieved (IRB-No: 2019-428-ProspDEuA). The present work adheres to the standards for reporting observational studies (STROBE) [12].

### 2.2. Tumor Staging and Lymph Node Classification

The post-treatment pathologic reports of all enrolled patients were carefully re-reviewed so that the staging data in this study were grouped according to the TNM classification of malignant tumors 8th edition [6]. LNR was calculated as the number of positive LNs divided by the number of examined LNs (NELN). LODDS was calculated by the following formula: log[(number of positive LNs + 0.5)/(NELN—number of positive LNs + 0.5)]. The novel LN classification schemes were analyzed as both continuous and categorical variables. When used as categorical variables, cut-off values and resulting subcategories as suggested by 25 different studies for LNR [13,14,15,16,17,18,19,20,21,22,23,24,25,26,27,28,29,30,31,32,33,34,35,36,37] and 27 different studies for LODDS [15,16,17,18,19,20,21,22,23,26,29,32,33,34,36,37,38,39,40,41,42,43,44,45,46,47,48] were used. Suggested cut-off values published after 31st December 2019 were not taken into account in our analysis. 

### 2.3. Statistical Analysis

The relationship between the number of metastatic LNs and the two alternative LN classifications was initially explored with scatter plots. The area under the receiver operating characteristic curve (AUC) was then measured in order to estimate the accuracy of LODDS and LNR as continuous variables by using SPSS Statistics for Windows (IBM SPSS Statistics for Windows, Version 25.0. Armonk, MY, USA: IBM Corp.) The prognostic potential of the examined LN classifications, when utilized as categorical variables, was investigated by using a multivariate Cox-regression model. The base model included the following covariates: age, sex, T category (T1 + 2, T3 + 4), grading (G1 + 2, G3 + 4), localization (head + corpus, tail), presence of metastatic disease, positivity of resection margins, and neoadjuvant therapy. Using this base model, we estimated the hazard ratios (HR) for each LN classification and evaluated model discrimination by means of C-statistics as recently described [49,50]. The difference between the C-index of the model including the N category and any other model of alternative LN classifications was compared by using the same data set and calculating the jackknife variance estimates of their difference. This difference was quantified by calculating the Delta C, and *p*-values (Pc) were adjusted by using the false discovery rate (FDR) method. Various subgroups of our patient collective were further explored as described above. These subgroups were defined by tumor localization (head/corpus, tail), presence of metastatic disease, and resection margins. Finally, we created a nomogram from 125 randomly selected patients based on a model that included the covariates that reached a *p* < 0.1 in the base model and the best LN classification and assessed the discriminatory power of this final model. Validation of the final model was performed by Bootstrap resampling (B = 100 times) based on our data set and assessing the calibration curves. A simple imputation method using the most common frequency for categorical values and medians for continuous values was used for risk factors with missing data. The statistical software R version 3.6.3 was used [51]. We used reporting tools based on the R package ‘knitr’ [52]. The R packages ‘survival’ and ‘rms’ were used for the analysis of the cox regression, estimation of the C-statistics, and construction of graphs [53,54].

## 3. Results

A total of 319 patients with PDAC were included in our study. The study population consisted of 174 (55%) males and 145 (45%) females. The median age was 68 years (range 17–95 years). The most common surgical procedure performed was partial pancreaticoduodenectomy (*n* = 274, 85.9%) followed by distal pancreas resection (*n* = 28, 8.8%) and total pancreatectomy (*n* = 17, 5.3%). In the majority of cases (*n* = 303, 95%) patients underwent upfront surgery without neoadjuvant therapy. Adjuvant therapy was completed in 263 (94.9%) cases. Baseline clinicopathological characteristics are presented in Table 1. 

**Figure 1:**
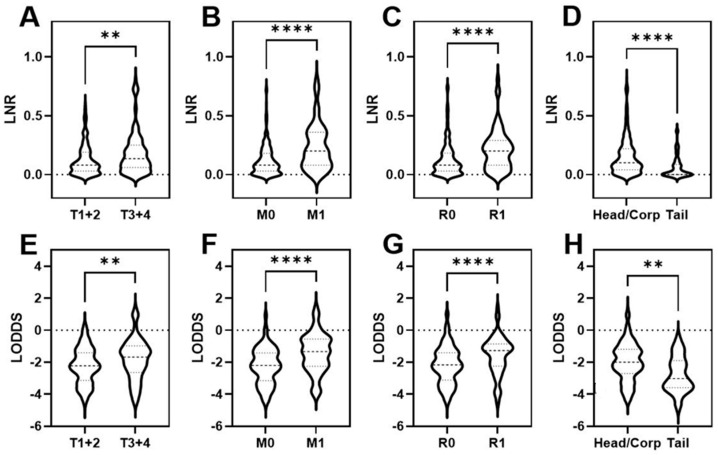
Violin plots depicting the association of LNR and LODDS with T category (**A**,**E**), presence of distant metastasis (**B**,**F**), resection margins (**C**,**G**), and tumor localization (**D**,**H**). ** *p* < 0.01; **** *p* < 0.0001.

Lymph node involvement was noted in 80.5% (*n* = 257) of the included patients. The median total harvested LNs (tLN) was 27 (range: 1–95). The median positive harvested LNs (pLN) were found to be 3 (range: 1–43). First, we investigated whether LNR or LODDS were associated with clinicopathological variables in patients with PDAC. Interestingly, advanced tumor stage, distant metastasis at time of surgery, positive resection margins, and PDAC of the head or corpus were associated with both increasing LNR and LODDS (Figure 1). In contrast, other variables such as age, grading, and sex did not differ significantly (data not shown). 

**Figure 2:**
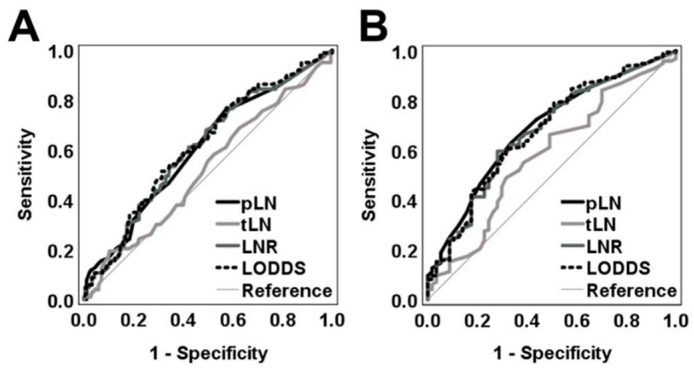
ROC analysis of various LN classification systems. ROC curves were generated for LNR, LODDS, pLN (positive lymph nodes), and tLN (total lymph nodes) as categorical variables to predict (**A**) 1-year OS and (**B**) 3-year OS.

Next, ROC curves were generated for pLN, tLN, LNR, and LODDS as continuous variables to predict 1- and 3-year OS (Figure 2A,B). The highest AUC values were demonstrated by the LODDS classification for both follow-up periods (Table S1).

**Figure 3:**
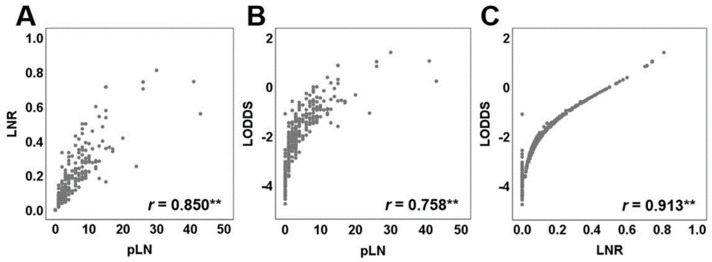
Relationship between positive lymph nodes (pLN), LNR, and LODDS. Scatter plots presenting the distribution of (**A**) LNR versus pLN, (**B**) LODDS versus pLN, and (**C**) LNR versus LODDS. ** *p* < 0.001.

Moreover, the relationship between pLN, LNR, and LODDS was further explored by means of scatter plots (Figure 3A–C). Both novel LN classifications demonstrated increasing values, parallel to pLN (rLODDS = 0.758, rLNR = 0.850). 

The prognostic performance was then examined for each N, LNR, and LODDS subcategory in the context of our base model (Tables S2–S4). The base model covariates (for the complete patient collective), and their prognostic values are listed in Table 2. 

Tumor localization, presence of metastatic disease, high tumor grading, and positivity of the resection margins were found to be independent risk factors for OS, in contrast to age, sex, and T category. The discriminative potential of the models including various LODDS and LNR classifications under investigation was further analyzed by C-statistics. Comparison of C-indices revealed superior discriminatory ability of distinct LNR and LODDS classifications compared to the N category. The results of C-statistic for the complete collective are presented in Tables S5 and S6.

The above-mentioned analysis of predictive power and discriminative potential was also performed in the following subgroups: cases with cancer of the pancreatic head and corpus only, cases with cancer of the pancreatic tail only, non-metastatic (M0) cases only, metastatic (M1) cases only, cases with positive resection margins (R1) only, and cases with negative resection margins (R0) only. Of note, a classification system is considered to be of practical clinical relevance when its subcategories demonstrate gradually increasing HRs, and this implies a reduced chance of OS for the higher subcategories. Moreover, the subcategories of the ideal LN classification system should all demonstrate statistical significance. LN classifications that were found to satisfy at least one of the two above-mentioned conditions are depicted in Table 3, Table 4 and Table 5.

The LODDS classification as described by Calero et al. [16] as well as the LNR classification as described by Arslan et al. [14] were the only alternative LN staging schemes that demonstrated statistically significant, gradually increasing HRs of their subcategories and at the same time significantly higher discriminatory potential compared to the N category, in the whole patient collective. LN classifications as proposed by other authors [35,40,47] only demonstrated the above-mentioned attributes in distinct patient subgroups. Of note, overall performance of the N category was not optimal, with the N1 subcategory being significantly important after Cox regression, namely, in the subgroup of patients with positive resection margins. Interestingly, no other alternative LN classification was found to entirely out-perform the N category in the patient subgroup with positive resection margins and the subgroup with disease localized in the pancreatic head or corpus. It should be noted, however, that the LODDS classification by Calero and colleagues [16], in contrast to the LNR classification by Arslan et al. [14], also exhibited a statistically significant higher C-index in the subgroup of M0 patients and therefore was implemented in our final model. 

**Figure 4:**
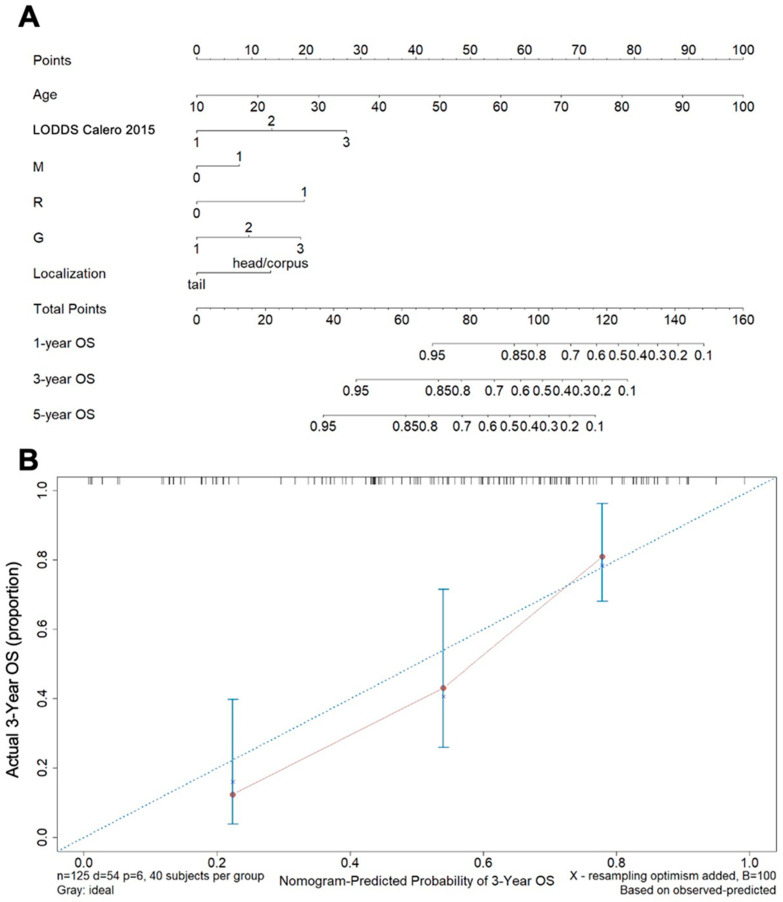
Nomogram for predicting patient survival probability. (**A**) A nomogram built out of the independent variables age, LODDS as reported by Calero et al. [16] presence of distant metastasis, resection margin status, differentiation and tumor localization predicting 1-, 3-, and 5-year OS. (**B**) The final model was validated by bootstrap resampling (B = 100 times) based on our data set and assessing the calibration curves.

Next, we constructed a nomogram from 125 randomly selected patients based on a final model including the five independent variables (age, localization, distant metastasis, differentiation, and resection margin) that reached a *p* < 0.1 in our multivariate base model and the best performing LN classification model (LODDS classification reported by Calero et al. [16]) that we obtained by Cox regression analysis as well as C-statistics (Figure 4A). According to this nomogram, a 51-year-old patient (45 points) with an R0-resection (0 points) and moderately differentiated (10) PDAC of the pancreatic head (12.5 points), LODDSCalero subcategory 314 (27.5 points) without distant metastasis (M0; 0 points) achieves a total of 95 points, which reflects a 1-, 3- and 5-year OS probability of 85%, 60%, and 45%, respectively. The C-index was 0.741 (SE 0.033), and the internal validation of our model by bootstrap resampling showed a parallel course of the curve to the diagonal ideal line, which underlines a close agreement between predicted and observed events.

## 4. Discussion

LN staging is of utmost importance for the classification, treatment strategy, and prognosis of the majority of solid tumors. The issue of stage migration gave rise to novel LN classification systems that attempt to more precisely stratify the cases in alternative patient subgroups. LNR was first introduced as a scheme that takes into account not only the amount of pLNs but also the extent of lymphadenectomy. Its main weakness is the inherent inability to further stratify cases that have pLN values of 0 or 1. This has been previously questioned and criticized [43]. To overcome this issue, LODDS was introduced as a novel classification that takes into account the extent of surgical radicality regarding lymphadenectomy, and it successfully substratifies cases with either no infiltrated LNs or infiltration of all harvested LNs. Both LNR and LODDS are continuous variables and have little clinical practicality when used as such. Therefore, various subcategories with distinct cut-off values need to be generated that express the advanced state of the tumor disease and also, ideally, have a prognostic significance.

The aim of the present study was to identify the most appropriate set of cut-off values for LNR and LODDS in patients with PDAC with regard to OS. Few studies have simultaneously analyzed the prognostic value of different LN classification schemes in cases with PDAC [43,55,56]. Of note, the prognostic power and discriminative ability of different cut-off values within distinct LNR and LODDS classifications has not yet been simultaneously assessed. To our knowledge, this is the first study to compare a large set of previously published LN classification systems in patients with PDAC, with regard to OS. After analyzing 25 different LNR and 27 LODDS classifications, we conclude that the LNR classification as proposed by Arslan et al. [14] and the LODDS classification as proposed by Calero et al. [16] both outperform the N category in terms of prognostic power and discriminative ability. More specifically, the LODDS classification of Calero et al. [16], when assessed in the context of the complete patient collective, pancreatic head/corpus only, non-metastatic, and negative resection margin cases demonstrated superiority regarding all above-mentioned attributes compared to the N category. This LN scheme was developed and evaluated in patients with gastric carcinoma and defines four LODDS subcategories by the following cut-off values: −3, −1, and 3. In our patient collective, the fourth patient subgroup defined by LODDS ≥ 3 did not include any patients because such values were not reached. Thus, the classification as originally described by Calero et al. [16] could be modified by omitting the fourth subgroup without losing its discriminative and predictive attributes, when applied to PDAC patients. Recently, we demonstrated the prognostic superiority of the LODDS classification proposed by Calero et al. [16] over the classical N category in patients with UICC Stage III colorectal cancer [49]. In addition, the LNR classification described by Arslan et al. [14], which consists of three subcategories as defined by the cut-off values of 0.05 and 0.2, outperformed the N category, not only within the whole patient collective but in the following subgroups as well: pancreatic head/corpus localization and patients with negative resection margins. However, although the LNR of Arslan et al. [14] demonstrated prognostic significance in patients with non-metastatic (M0) disease, its discriminatory power was not superior to the N category. Arslan and colleagues originally developed their LNR scheme for use in patients with node-negative colon cancer and failed to demonstrate a better performance in the original publication [14]. The performance of the investigated LN classifications within the complete patient collective is of special interest because it reflects their practical utilization potential in everyday clinical practice. The LODDS classification of Calero et al. [16] also better predicted the prognosis in the subgroups of radically resected cases and cases with disease localization in the pancreatic head or corpus. Of note, the decreased number of cases with pancreatic tail tumors (*n* = 28) may have hampered a meaningful statistical analysis within this subgroup of patients. Further novel LN classifications were found to outperform the N category in every aspect when analyzed in the subgroup of radically resected patients with negative resection margins, all of which were LODDS schemes [34,36,40,45,47]. 

The tLN has been shown to have a positive linear relationship with survival in patients undergoing resection for PDAC [57]. All of the cases included in this work were treated in a single tertiary referral center in a standardized manner. More specifically, emphasis is put on the extent of the lymphadenectomy. This is reflected by the amount of tLNs of our collective with a median of 27 resected LNs. In order for a LN classification scheme to be of comprehensive and reasonable use, apart from its design, it is a prerequisite that the quality of surgery be unquestionable. Adequate LN staging requires the maximal amount of harvested LNs. If this cannot be guaranteed, then LN schemes are of questionable value, and their clinical implementation is debatable. 

The choice of the most appropriate adjuvant chemotherapy regimen is not yet based on a standard treatment protocol [58,59]. More appropriate LN classifications could substratify cases with resected PDAC and aid the multi-disciplinary decision-making process regarding administration of combined chemotherapeutical agents. The concomitant administration of radiotherapy is also an issue that could be investigated by using novel LN schemes. Such an application of novel LN classification schemes has recently been explored in the work of Zhu et al. [60], in which adjuvant radiotherapy was found to improve survival in negative-margin patients with LNR values ranging from 0.15 to 0.25. Similar studies should be designed with focus on the utilization of novel LN classifications for a tailored approach regarding adjuvant chemoradiation, in line with our findings. 

Nevertheless, this study has some limitations. First, the retrospective study design had an inherent bias. The relatively small sample size is a further drawback of our work. Third, disease-free survival could not be maintained. On the other hand, the consistently radical surgery performed by dedicated pancreatic surgeons and the long duration of follow up compensate to a great extent and provide a comprehensive review of the current alternative LN classifications in PDAC. 

## 5. Conclusions

This is the first study in which various novel LN classification schemes were compared to the standard N category in patients with resected PDAC followed by adjuvant treatment, with regard to OS. In conclusion, our results demonstrate that especially the LODDS classification with cut-off values as proposed by Calero et al. [16] outperforms the standard N category in terms of predictive power and discriminative ability and lays the groundwork for future research, within the framework of large-scale clinical trials.

## Figures and Tables

**Table 1:** Patient characteristics.

Variable	All Patients (%)	PPPD (%)	Distal Pancreatectomy (%)	Total Pancreatectomy (%)
**Number of subjects**	319	274	28	17
**Age**				
Median (range)	68 (17–95)	69 (42–95)	61.5 (17–80)	70 (55–83)
**Sex**				
Male	174 (55)	150 (54.7)	16 (57.1)	8 (47.1)
Female	145 (45)	124 (45.3)	12 (42.9)	9 (52.9)
**Localization**				
Head/corpus	291 (91)			
Tail	28 (8.8)			
**Extend of tumor (T)**				
T1	25 (7.8)	21 (7.7)	4 (14.3)	0
T2	166 (52)	152 (55.2)	9 (32.1)	5 (29.4)
T3	120 (37.6)	95 (34.7)	15 (53.6)	10 (58.8)
T4	8 (2.6)	6 (2.2)	0	2 (11.8)
**Lymph node metastasis (N)**				
N0	62 (19.4)	45 (16.4)	15 (53.6)	2 (11.8)
N1	241 (75.5)	219 (79.9)	13 (46.4)	9 (52.9)
N2	16 (5.1)	10 (3.6)	0	6 (35.3)
**tLN median (range)**	27 (1–95)	27 (6–95)	17.5 (1–58)	42 (13–68)
**pLN median (range)**	3 (0–43)	3 (0–43)	0 (0–13)	4 (0–20)
**Distant metastasis (M)**				
M0	248 (78)	218 (79.6)	20 (71.4)	10 (58.8)
M1	71 (22)	56 (20.4)	8 (28.6)	7 (41.2)
**Resection margin (R)**				
R0	254 (70)	220 (80.3)	24 (85.7)	10 (58.8)
R1	65 (20)	54 (19.7)	4 (14.3)	7 (41.2)
**Differentiation (G)**				
G1	4 (1.3)	1 (0.4)	3 (10.7)	0
G2	180 (56.4)	153 (55.8)	16 (57.1)	11 (64.7)
G3	135 (42.3)	120 (43.8)	9 (32.1)	6 (35.3)
**Neoadj. therapy**				
Yes	16 (5)	8 (2.9)	6 (21.4)	2 (11.8)
No	303 (95)	266 (97.1)	22 (78.6)	15 (88.2)
**Adjuvant therapy**				
Yes	263/277 (94.9)	224/237 (94.5)	25/26 (96.1)	14 (82.4)
No	14/277 (5.1)	10/237 (5.5)	1/26 (3.9)	3 (17.6)
Unknown	42 (13.2)	40 (14.6)	2 (7.1)	0

Abbreviations: LN = lymph node, tLN = total amount of harvested LNs, pLN = positive LNs, PPPD = partial pylorus preserving pancreatoduodenectomy, Neoadj. = neoadjuvant.

**Table 2:** Cox regression analysis of the variables considered for the multi-variable adjusted base model.

Clinicopathological Variable	HR (95% CI)	*p* Value
**Sex**		
Female	1.00 (reference)	0.458
Male	1.11 (0.85–1.44)	
**Age**		
<68	1.00 (reference)	0.052
≥68	1.31 (1.00–1.72)	
**Localization**		
Tail	1.00 (reference)	**0.003**
Head/corpus	2.50 (1.37–4.58)	
**Neoadjuvant therapy**		
No	1.00 (reference)	0.344
Yes	1.41 (0.69–2.88)	
**Extend of tumor**		
T1 + 2	0.92 (0.70–1.22)	0.566
T3 + 4	1.00 (reference)	
**Distant metastasis**		
M0	1.00 (reference)	**0.003**
M1	1.63 (1.19–2.24)	
**Differentiation**		
G1 + 2	0.65 (0.50–0.85)	**0.002**
G3 + 4	1.00 (reference)	
**Resection margins**		
R0	1.00 (reference)	**0.024**
R1	1.44 (1.05–1.99)	

**Table 3:** Presentation of LNR classifications and their statistical significance (YES/NO) with regard to C statistic (p_c_), Cox regression of all subcategories (p_HR_), and ascending values of HRs parallel to the assorted risk groups (Incr. HR) for various patient subgroups. Classifications that satisfy an investigated parameter are marked blue, and classifications that satisfy all three investigated parameters are marked green.

	All Cases			Head/Corpus			Tail			M(0)			M(+)			R(0)			R(+)		
LNR	p_c_ <0.05	p_HR_ <0.05	Incr. HR	p_c_ <0.05	p_HR_ <0.05	Incr. HR	p_c_ <0.05	p_HR_ <0.05	Incr. HR	p_c_ <0.05	p_HR_ <0.05	Incr. HR	p_c_ <0.05	p_HR_ <0.05	Incr. HR	p_c_ <0.05	p_HR_ <0.05	Incr. HR	p_c_ <0.05	p_HR_ <0.05	Incr. HR
Agnes [13]	NO	NO	NO	NO	NO	NO	NO	NO	NO	NO	NO	YES	NO	NO	NO	NO	NO	NO	YES	NO	NO
Arslan [14]	YES	YES	YES	YES	YES	YES	NO	NO	NO	NO	YES	YES	NO	NO	NO	YES	YES	YES	NO	NO	NO
Bagante [15]	NO	NO	YES	NO	NO	YES	NO	NO	NO	NO	NO	YES	NO	NO	NO	NO	NO	NO	NO	NO	NO
Calero [16]	NO	NO	NO	NO	NO	YES	NO	NO	NO	NO	NO	YES	NO	NO	NO	NO	NO	NO	NO	NO	NO
Cao [17]	NO	NO	NO	NO	NO	NO	NO	NO	NO	NO	NO	NO	NO	NO	NO	NO	NO	NO	NO	NO	NO
Chang [18]	NO	YES	NO	YES	YES	NO	NO	NO	NO	NO	YES	NO	NO	NO	NO	YES	NO	NO	NO	NO	NO
Conci [19]	NO	NO	NO	NO	NO	NO	NO	NO	NO	NO	NO	YES	NO	NO	NO	NO	NO	NO	NO	YES	NO
Fang [20]	NO	YES	YES	NO	YES	YES	NO	NO	NO	NO	YES	YES	NO	NO	NO	NO	YES	YES	NO	NO	NO
Fortea-S. [21]	NO	NO	YES	NO	NO	YES	NO	NO	NO	NO	NO	YES	NO	NO	NO	NO	NO	YES	NO	NO	NO
Huang [22]	NO	NO	NO	NO	NO	NO	NO	NO	NO	NO	NO	NO	NO	NO	NO	NO	NO	NO	NO	NO	NO
Jian-Hui [23]	NO	NO	NO	NO	NO	YES	NO	NO	NO	NO	NO	YES	NO	NO	NO	NO	NO	YES	NO	NO	NO
Kim [24]	NO	NO	NO	NO	NO	YES	NO	NO	NO	NO	NO	NO	NO	NO	NO	NO	NO	NO	NO	YES	YES
La Torre [25]	NO	NO	YES	NO	NO	YES	NO	NO	NO	NO	NO	YES	NO	NO	NO	NO	NO	NO	NO	NO	NO
Lee [26]	NO	NO	NO	NO	NO	NO	NO	NO	NO	NO	NO	NO	NO	NO	NO	NO	NO	NO	NO	NO	NO
Liu [27]	NO	NO	YES	NO	NO	YES	NO	NO	NO	NO	NO	YES	NO	NO	NO	NO	NO	NO	YES	NO	NO
Malleo [28]	NO	NO	YES	NO	NO	YES	NO	NO	NO	NO	NO	YES	NO	NO	NO	NO	NO	NO	YES	NO	NO
Riediger [29]	NO	YES	NO	NO	YES	YES	NO	NO	NO	NO	YES	YES	NO	NO	NO	NO	YES	NO	NO	NO	NO
Rosenberg [30]	NO	NO	YES	NO	NO	YES	NO	NO	NO	NO	NO	YES	NO	NO	NO	NO	NO	NO	YES	NO	NO
Smith [31]	NO	NO	NO	NO	NO	NO	NO	NO	NO	NO	NO	NO	NO	NO	NO	NO	NO	NO	NO	NO	NO
Song [32]	NO	NO	NO	NO	NO	NO	NO	NO	NO	NO	NO	YES	NO	NO	NO	NO	NO	NO	NO	NO	NO
Sun [33]	NO	NO	YES	NO	NO	YES	NO	NO	NO	NO	NO	YES	NO	NO	NO	NO	NO	NO	NO	YES	NO
Wang [34]	NO	NO	NO	NO	NO	YES	NO	NO	NO	NO	YES	YES	NO	NO	NO	NO	NO	YES	NO	NO	NO
Wang [35]	NO	NO	NO	NO	NO	NO	NO	NO	NO	NO	NO	YES	NO	NO	NO	NO	NO	NO	NO	NO	NO
Xu [36]	NO	NO	YES	NO	NO	YES	NO	NO	NO	NO	NO	YES	NO	NO	NO	NO	NO	NO	YES	NO	NO
Zhou [37]	NO	NO	NO	NO	NO	NO	NO	NO	NO	NO	NO	YES	NO	NO	NO	NO	NO	NO	NO	NO	NO

**Table 4:** Presentation of LODDS classifications and their statistical significance (YES/NO) with regard to C statistic (p_c_), Cox regression (p_HR_), and ascending values of HRs parallel to the assorted risk groups (Incr. HR) for various patient subgroups. Classifications that satisfy an investigated parameter are marked blue, and classifications that satisfy all three investigated parameters are marked green.

	All Cases			Head/Corpus			Tail			M(0)			M(+)			R(0)			R(+)		
LODDS	p_c_ <0.05	p_HR_ <0.05	Incr. HR	p_c_ <0.05	p_HR_ <0.05	Incr. HR	p_c_ <0.05	p_HR_ <0.05	Incr. HR	p_c_ <0.05	p_HR_ <0.05	Incr. HR	p_c_ <0.05	p_HR_ <0.05	Incr. HR	p_c_ <0.05	p_HR_ <0.05	Incr. HR	p_c_ <0.05	p_HR_ <0.05	Incr. HR
Amini [38]	NO	YES	YES	NO	YES	YES	NO	NO	NO	NO	YES	YES	NO	NO	NO	NO	YES	YES	NO	NO	NO
Amini [39]	NO	YES	YES	NO	YES	YES	NO	NO	NO	NO	YES	YES	NO	NO	NO	NO	YES	YES	NO	NO	NO
Bagante [15]	NO	NO	NO	NO	YES	NO	NO	NO	NO	NO	YES	NO	NO	NO	NO	NO	YES	YES	NO	NO	NO
Calero [16]	YES	YES	YES	YES	YES	YES	NO	NO	YES	YES	YES	YES	NO	NO	NO	YES	YES	YES	NO	NO	NO
Cao [40]	YES	NO	NO	YES	YES	YES	NO	NO	NO	YES	YES	YES	NO	NO	NO	YES	YES	YES	NO	NO	NO
Cao [17]	NO	NO	NO	NO	NO	NO	NO	NO	NO	NO	NO	NO	NO	NO	NO	NO	NO	NO	NO	NO	NO
Chang [18]	NO	NO	NO	NO	NO	NO	NO	NO	NO	NO	NO	YES	NO	NO	NO	NO	NO	NO	NO	NO	NO
Conci [19]	NO	NO	NO	NO	NO	NO	NO	NO	NO	NO	NO	YES	NO	NO	NO	NO	NO	NO	NO	NO	NO
Fang [20]	NO	NO	NO	NO	NO	NO	NO	NO	NO	NO	NO	YES	NO	NO	NO	NO	NO	NO	NO	NO	NO
Fortea-S. [21]	NO	YES	YES	NO	YES	YES	NO	NO	YES	NO	YES	YES	NO	NO	NO	NO	YES	YES	NO	NO	NO
He [41]	YES	NO	NO	YES	NO	NO	NO	NO	NO	YES	NO	YES	NO	NO	NO	YES	NO	YES	NO	NO	NO
Huang [22]	NO	NO	NO	NO	NO	NO	NO	NO	NO	NO	NO	NO	NO	NO	NO	NO	NO	NO	NO	NO	NO
Jian-Hui [23]	NO	NO	NO	NO	NO	YES	NO	NO	NO	NO	NO	YES	NO	NO	NO	NO	NO	NO	NO	NO	NO
Lee [26]	NO	NO	NO	NO	NO	NO	YES	NO	NO	NO	NO	YES	NO	NO	NO	YES	NO	NO	NO	NO	NO
Persiani [42]	NO	NO	NO	NO	NO	NO	NO	NO	NO	NO	NO	YES	NO	NO	NO	NO	NO	NO	NO	NO	NO
Ramacciato [43]	NO	NO	NO	NO	NO	NO	NO	NO	NO	NO	NO	NO	NO	NO	NO	NO	NO	NO	NO	NO	NO
Riediger [29]	NO	NO	NO	NO	NO	NO	NO	NO	NO	NO	NO	NO	NO	NO	NO	NO	NO	NO	NO	NO	NO
Song [32]	NO	NO	NO	NO	NO	NO	NO	NO	NO	YES	YES	NO	NO	NO	NO	NO	YES	NO	NO	NO	NO
Sun [33]	NO	NO	NO	NO	NO	NO	NO	NO	NO	NO	NO	NO	NO	NO	NO	NO	NO	NO	NO	NO	NO
Toth [44]	NO	NO	YES	NO	NO	YES	NO	NO	NO	NO	NO	YES	NO	NO	NO	NO	NO	NO	NO	NO	NO
Wang [45]	NO	NO	NO	NO	NO	NO	NO	NO	NO	NO	YES	YES	NO	NO	NO	YES	YES	YES	NO	NO	NO
Wang [35]	NO	NO	YES	NO	NO	YES	NO	NO	YES	NO	NO	YES	NO	NO	NO	NO	NO	YES	NO	NO	NO
Wu [46]	NO	NO	NO	NO	NO	NO	NO	NO	NO	NO	NO	NO	NO	NO	NO	NO	NO	YES	NO	NO	NO
Xu [36]	NO	NO	NO	NO	NO	NO	NO	NO	NO	NO	NO	NO	NO	NO	NO	NO	NO	NO	NO	NO	NO
Xu [47]	NO	YES	YES	NO	YES	YES	NO	NO	NO	YES	NO	YES	NO	NO	NO	YES	YES	YES	NO	NO	NO
Yang [48]	NO	NO	NO	NO	NO	NO	NO	NO	NO	NO	NO	NO	NO	NO	NO	NO	NO	NO	NO	NO	NO
Zhou [37]	NO	NO	NO	NO	NO	NO	NO	NO	NO	NO	NO	YES	NO	NO	NO	NO	NO	NO	NO	NO	NO

**Table 5:** Presentation of N category and its statistical significance (YES/NO) with regard to Cox regression (p_HR_) and ascending values of Hazard Ratios parallel to the assorted risk groups (Incr. HR) for various patient subgroups. Classifications that satisfy all investigated parameters are marked green.

	All Cases			Head/Corpus			Tail			M(0)			M(+)			R(0)			R(+)		
	p_c_ <0.05	p_HR_ <0.05	Incr. HR	p_c_ <0.05	p_HR_ <0.05	Incr. HR	p_c_ <0.05	p_HR_ <0.05	Incr. HR	p_c_ <0.05	p_HR_ <0.05	Incr. HR	p_c_ <0.05	p_HR_ <0.05	Incr. HR	p_c_ <0.05	p_HR_ <0.05	Incr. HR	p_c_ <0.05	p_HR_ <0.05	Incr. HR
**N category**	(Ref.)	NO	NO	(Ref.)	NO	NO	(Ref.)	NO	YES	(Ref.)	NO	NO	(Ref.)	NO	NO	(Ref.)	NO	NO	(Ref.)	NO	YES

## Data Availability

The data presented in this study are available on request from the corresponding author. The data are not publicly available due to ethical issues.

## Supplementary Materials

The following supporting information can be downloaded at: https://www.mdpi.com/article/10.3390/cancers14071834/s1. Table S1: ROC analysis of various LN classification systems for 1-year and 3-year OS. Table S2: OS depending on the N classification. Table S3: OS depending on the respective LNR classification. Each LNR subgroup is defined by a LNR range as indicated. Table S4: OS depending on the respective LODDS classification. Each LODDS subgroup is defined by a LODDS range as indicated. * The indicated subgroups were omitted as none of our included patients exhibited LODDS value within this range. Table S5: C Statistic as a measure of model discrimination for various LNR classifications in comparison with the N category for the entire patient collective. Table S6: C Statistic as a measure of model discrimination for various LODDS classifications in comparison with the N category for the entire patient collective.
