# Supplementary material for: N, LNR or LODDS: Which Is the Most Appropriate Lymph Node Classification Scheme for Patients with Radically Resected Pancreatic Cancer?
Source: Cancers (Basel). 2022 Apr 6;14(7):1834. doi: 10.3390/cancers14071834 (PMC8997819; doi:10.3390/cancers14071834)
Supplement: Supplementary file 1 [file cancers-14-01834-s001.zip › cancers-1645706-Supplementary.pdf]

Table S1. ROC analysis of the total number of resected lymph nodes (tLN), positive lymph nodes (pLN), LNR and LODDS. for 1- year, and 3-year OS.

**Overall Survival (OS)**	**LN evaluation**	**ROC analysis**		
	**Type**	**AUC**	**95% CI**	**P value**
**1-year OS**	LODDS	0.616	0.549-0.683	**0.001**
	LNR	0.608	0.540-0.676	**0.002**
	tLN	0.521	0.451-0.591	0.558
	pLN	0.605	0.537-0.673	**0.003**
**3-year OS**	LODDS	0.676	0.596-0.755	**>0.001**
	LNR	0.672	0.593-0.751	**>0.001**
	tLN	0.579	0.494-0.664	0.068
	pLN	0.686	0.610-0.763	**>0.001**

Table S2 OS depending on the N classification

	**Subcategory**	**HR***	**95%CI***	**P value***
N category	0	1	(Reference)	
	1	1.233	0.871-1.744	0.237
	2	0.638	0.193-2.109	0.461

*Adjusted for sex, age, localization, neoadjuvant therapy, extend of tumor (T), distant metastasis (M), differentiation (G) and resection margin (R) using the base model

Table S3 OS depending on the respective LNR classification. Each LNR subcategory is defined by a LNR range as indicated

**LNR classification**	**Subcategory**	**LNR Range**	**HR***	**95%CI***	**P value***
Agnes et al. [13]	1	0	1	(reference)	
	2	0.01; 0.10	0.947	0.626-1.432	0.795
	3	0.11; 0.25	1.472	0.995-2.178	0.053
	4	0.26; 0.40	1.227	0.743-2.026	0.425
	5	> 0.40	2.484	1.379-4.475	**0.002**
Arslan et al. [14]	1	≤ 0.05	1	(reference)	
	2	> 0.05-0.20	1.563	1.107-2.207	**0.011**

	3	> 0.20	1.697	1.155-2.494	**0.007**
Bagante et al. [15]	1	0	1	(reference)	
	2	0.01; 0.25	1.176	0.821-1.685	0.378
	3	0.26; 0.50	1.357	0.846-2.176	0.205
	4	> 0.50	2.097	1.008-4.361	**0.047**
Calero et al. [16]	1	0	1	(reference)	
	2	0.01; 0.25	1.170	0.816-1.676	0.393
	3	0.26; 0.75	1.447	0.918-2.280	0.112
	4	> 0.75	2.990	0.382-23.350	0.297
Cao et al. [17]	1	0; 0.24	1	(reference)	
	2	0.25; 0.28	1.210	0.846-1.729	0.296
	3	> 0.28	1.254	0.781-2.015	0.349
Chang et al. [18]	1	≤ 0.08	1	(reference)	
	2	0.09; 0.17	1.911	1.343-2.722	**0.0003**
	3	0.18; 0.33	1.466	1.027-2.029	**0.035**
	4	> 0.33	1.660	1.040-2.651	**0.034**
Conci et al. [19]	1	0	1	(reference)	
	2	> 0; ≤ 0.25	1.174	0.818-1.683	0.384
	3	> 0.25	1.392	0.892-2.173	0.146
Fang et al. [20]	1	< 0.10	1	(reference)	
	2	0.10; 0.33	1.532	1.139-2.060	**0.005**
	3	≥ 0.34	1.590	1.004-2.519	**0.048**
Fortea-Sanchis et al. [21]	1	0; 0.24	1	(reference)	
	2	0.25; 0.60	1.171	0.822-1.667	0.381
	3	> 0.60	2.487	1.038-5.964	**0.041**
Huang et al. [22]	1	< 0.25	1	(reference)	
	2	≥ 0.25; < 0.5	1.104	0.757-1.611	0.607
	3	≥ 0.50; < 0.75	1.939	0.983-3.828	0.056
	4	≥ 0.75	1.485	0.451-4.894	0.516
Jian-Hui et al. [23]	1	0	1	(reference)	
	2	0.01; ≤ 0.10	0.920	0.609-1.390	0.692
	3	> 0.10; ≤ 0.25	1.462	0.988-2.164	0.058
	4	> 0.25	1.520	0.967-2.390	0.070
La Torre et al. [25]	1	0	1	(reference)	
	2	0.010; 0.199	1.170	0.808-1.692	0.405
	3	0.200; 0.399	1.238	0.803-1.909	0.335
	4	> 0.399	2.233	1.247-4.000	**0.007**
Lee et al. [26]	1	≤ 0.10	1	(reference)	
	2	>0.10; ≤ 0.20	1.566	1.110-2.208	**0.011**
	3	>0.20; ≤ 0.30	1.687	1.123-2.533	**0.012**
	4	>0.30	1.380	0.900-2.117	0.139
Liu et al. [27]	1	0	1	(reference)	

	2	0.01; 0.10	0.943	0.624-1.426	0.781
	3	0.11; 0.40	1.405	0.962-2.052	0.078
	4	> 0.40	2.506	1.392-4.512	**0.002**
Malleo et al. [28]	1	0	1	(reference)	
	2	>0; ≤0.20	1.169	0.809-1.690	0.405
	3	>0.20; 0.40	1.236	0.800-1.909	0.339
	4	>0.40	2.496	1.367-4.459	**0.003**
Negi et al. [24]	1	0	1	(reference)	
	2	> 0; ≤ 0.50	1.206	0.848-1.716	0.298
	3	> 0.50	2.061	0.992-4.283	0.053
Riediger et al. [29]	1	< 0.10	1	(reference)	
	2	0.10; 0.199	1.583	1.132-2.215	**0.007**
	3	≥ 0.20	1.499	1.064-2.113	**0.021**
Rosenberg et al. [30]	1	0	1	(reference)	
	2	0.01; 0.17	1.176	0.805-1.716	0.401
	3	0.18; 0.41	1.224	0.813-1.842	0.333
	4	0.42; 0.69	1.188	1.114-4.298	**0.023**
	5	≥0.70	2.933	1.155-7.446	**0.024**
Smith et al. [31]	1	0	1	(reference)	
	2	>0; ≤ 1/15	0.835	0.513-1.360	0.469
	3	>1/15; ≤ 3/10	1.405	0.673-2.028	0.070
	4	>3/10; ≤ 7/10	1.156	0.697-1.914	0.574
	5	> 7/10	2.861	1.123-7.284	**0.027**
Song et al. [32]	1	0	1	(reference)	
	2	0.01; 0.11	1.005	0.673-1.499	0.981
	3	0.12; 0.36	1.485	1.011-2.181	**0.044**
	4	0.37; 0.66	1.130	0.603-2.118	0.703
	5	> 0.66	3.015	1.184-7.679	**0.020**
Sun et al. [33]	1	0	1	(reference)	
	2	0.01; 0.20	1.151	0.797-1.662	0.454
	3	0.21; 0.50	1.347	0.883-2.055	0.166
	4	> 0.5	2.085	1.004-4.331	**0.049**
Wang et al. [34]	1	0	1	(reference)	
	2	0.01; 0.30	1.475	1.075-2.025	**0.016**
	3	0.31; 0.60	1.398	0.913-2.141	0.123
	4	0.61; 1	2.327	1.224-4.425	**0.001**
Wang et al. [35]	1	< 0.07	1	(reference)	
	2	0.07; < 0.25	1.228	0.860-1.753	0.258
	3	0.25; < 0.50	1.149	0.691-1.910	0.592
	4	0.50; 1	2.777	1.092-7.065	**0.032**
Xu et al. [36]	1	0	1	(reference)	
	2	>0; ≤ 0.125	1.072	0.723-1.589	0.729

	3	> 0.125; ≤ 0.425	1.322	0.896-1.951	0.159
	4	> 0.425; ≤ 1	2.400	1.307-4.403	**0.005**
Zhou et al. [37]	1	0; ≤0.30	1	(reference)	
	2	>0.30; ≤0.70	0.971	0.642-1.468	0.889
	3	>0.70; ≤1	2.290	0.957-5.474	0.063

*Adjusted for sex, age, localization, neoadjuvant therapy, extend of tumor (T), distant metastasis (M), differentiation (G) and resection margin (R) using the base model

Table S4 OS depending on the respective LODDS classification. Each LODDS subcategory is defined by a value range as indicated. *The indicated LODDS subcategories were omitted as none of our included patients exhibited LODDS value within this range

LODDS classification	Subcategory	LODDS Range	HR*	95%CI*	P value*
Amini et al. [38]	1	≤ -2	1	(Reference)	
	2	-2; < 0	1.499	1.118-2.012	**0.007**
	3	≥ 0	2.224	1.193-4.150	**0.012**
Amini et al. [39]	1	≤ -3	1	(Reference)	
	2	> -3; < 0	1.660	1.168-2.358	**0.005**
	3	≥ 0	2.742	1.396-5.388	**0.003**
Bagante et al. [15]	1	< -2		(Reference)	
	2	- 1.99; -0.90	1.607	1.187-2.178	**0.002**
	3	- 0.89; 1.50	1.390	0.924-2.093	0.114
	4*	> 1.5	-	-	-
Calero et al. [16]	1	≤ -3	1	(Reference)	
	2	>-3; ≤-1	1.649	1.153-2.358	**0.006**
	3	>-1; ≤3	1.837	1.173-2.878	**0.008**
	4*	>3	-	-	-
Cao et al. [17]	1	≤ -0.5	1	(Reference)	
	2	> -0.5; ≤ 0	0.842	0.446-1.589	0.595
	3	> 0; ≤ 0.5	1.261	0.464-3.428	0.650
	4	> 0.5	2.256	0.949-5.366	0.066
Cao et al. [40]	1	≤ -2.6	1	(Reference)	
	2	> -2.6; ≤ -1.6	1.573	1.088-2.274	**0.016**
	3	> -1.6; ≤ -0.5	1.725	1.201-2.478	**0.003**
	4	> -0.5	1.679	0.961-2.934	0.069
Chang et al. [18]	1	≤ -0.92	1	(Reference)	
	2	-0.91; -0.62	0.982	0.560-1.723	0.949
	3	-0.61; -0.26	0.816	0.453-1.468	0.497
	4	> -0.26	2.049	1.194-3.517	**0.009**
Conci et al. [19]	1	≤ -1.35	1	(Reference)	
	2	> -1.35; ≤ -1	1.223	0.789-1.896	0.368
	3	> -1; ≤ -0.25	1.082	0.718-1.630	0.706

	4	> -0.25	2.197	1.273-3.792	**0.005**
Fang et al. [20]	1	< -0.82	1	(Reference)	
	2	≥ -0.82; < -0.57	0.895	0.525-1.526	0.684
	3	≥ -0.57	1.118	0.691-1.811	0.649
Fortea-Sanchis et al. [21]	1	< -2	1	(Reference)	
	2	≥ -2; ≤ -1	1.541	1.129-2.104	**0.006**
	3	> -1	1.561	1.064-2.291	**0.023**
He et al. [41]	1	< -3	1	(Reference)	
	2	≥ -3; < -2	1.438	0.943-2.192	0.092
	3	≥ -2; < -1	1.836	1.251-2.697	**0.002**
	4	≥ -1; < 0	1.688	1.048-2.717	**0.031**
	5	≥ 0	2.723	1.388-5.345	**0.004**
Huang et al. [22]	1	< -1	1	(Reference)	
	2	≥ -1; < 0	1.124	0.764-1.655	0.551
	3	≥ 0; < 1	1.945	0.985-3.838	0.055
	4	≥ 1; < 2	1.495	0.454-4.929	0.509
	5*	≥ 2	-	-	
Jian-Hui et al. [23]	1	≤ -1.5	1	(Reference)	
	2	> -1.5; ≤ -1	1.241	0.869-1.772	0.236
	3	> -1; ≤ 0	1.247	0.842-1.847	0.270
	4	> 0	1.935	0.987-3.792	0.055
Lee et al. [26]	1	≤ -4	1	(Reference)	
	2	> -4; ≤ -2.5	1.042	0.471-2.307	0.919
	3	> -2.5; ≤ -2	1.394	0.609-3.194	0.432
	4	> -2; ≤ -0.5	1.763	0.814-3.818	0.150
	5	> -0.5	1.682	0.690-4.101	0.253
Persiani et al. [42]	1	≤ -1.36	1	(Reference)	
	2	-1.35; -0.53	1.297	0.935-1.800	0.120
	3	≥ -0.52	1.178	0.711-1.953	0.525
Ramacciato et al. [43]	1	<-0.005	1	(Reference)	
	2	≥ -0.005; <0.012	1.893	0.460-7.795	0.377
	3*	≥ 0.012; < 0.026	-	-	-
	4	≥ 0.026	1.744	0.905-3.361	0.097
Riediger et al. [29]	1	< -1	1	(Reference)	
	2	-1; -0.500	1.276	0.847-1.923	0.244
	3	-0.499; -0.001	0.777	0.384-1.573	0.483
	4	≥ 0	1.789	0.974-3.285	0.061
	5*	> -0.5	-	-	-
Song et al. [32]	1	≤ -2.51	1	(Reference)	
	2	> -2.51; ≤ -1.68	1.610	1.107-2.343	0.013
	3	> -1.68; ≤ -0.51	1.678	1.188-2.370	**0.003**
	4	> -0.51; ≤ 0.73	1.365	0.745-2.499	0.314

	5	> 0.73	3.552	1.430-8.825	**0.006**
Sun et al. [33]	1	≤ -1.500	1	(Reference)	
	2	-1.499; -1.000	1.231	0.862-1.759	0.253
	3	-0.999; -0.500	1.376	0.900-2.104	0.141
	4	-0.499; 0	0.959	0.497-1.850	0.900
	5	> 0	1.901	0.969-3.730	0.062
Toth et al. [44]	1	< -1.125	1	(Reference)	
	2	-1.125; -0.251	1.041	0.722-1.501	0.830
	3	-0.250; 0.749	1.972	1.025-3.792	**0.042**
	4	≥ 0.750	2.450	1.026-5.851	**0.044**
Wang et al. [45]	1	< -2.2	1	(Reference)	
	2	-2.2; < -1.1	1.463	1.069-2.002	**0.017**
	3	-1.1; < 0	1.340	0.901-1.994	0.148
	4	0; < 1.1	2.191	1.138-4.218	**0.019**
	5	≥ 1.1	3.314	0.429-25.595	0.251
Wang et al. [35]	1	≤ -2.5	1	(Reference)	
	2	> -2.5; ≤ -1	1.573	1.143-2.165	**0.005**
	3	> -1; ≤ 0.9	1.666	1.101-2.520	**0.016**
	4	> 0.9	2.165	0.640-7.327	0.214
Wu et al. [46]	1	≤ -1.46	1	(Reference)	
	2	- 1.45; -1.17	1.409	0.925-2.147	0.110
	3	-1.16; - 0.73	1.386	0.900-2.137	0.139
	4	≥ -0.72	1.357	0.889-2.069	0.157
Xu et al. [36]	1	≤ -2.80	1	(Reference)	
	2	> -2.80; ≤ -1.60	1.509	1.035-2.201	**0.033**
	3	> -1.60; ≤ -0.31	1.595	1.087-2.339	**0.017**
	4	> -0.31	2.965	1.632-5.387	**0.0004**
Xu et al. [47]	1	< -1	1	(Reference)	
	2	-1.000; -0.501	1.294	0.857-1.952	0.220
	3	-0.500; -0.001	0.796	0.392-1.617	0.528
	4	0; 0.499	1.452	0.636-3.314	0.376
	5	≥ 0.500	2.396	0.998-5.756	0.051
Yang et al. [48]	1	≤ -1.43	1	(Reference)	
	2	> -1.43; ≤ -1.20	1.447	0.898-2.332	0.129
	3	> -1.20; ≤ -0.69	1.356	0.912-2.018	0.133
	4	> -0.69	1.307	0.853-2.003	0.219
Zhou et al. [37]	1	≤ -1	1	(Reference)	
	2	-0.999; -0.500	1.317	0.874-1.985	0.188
	3	-0.499; 0.500	0.990	0.566-1.733	0.973
	4	> 0.500	2.468	1.028-5.927	**0.043**

*Adjusted for sex, age, localization, neoadjuvant therapy, extend of tumor (T), distant metastasis (M), differentiation (G) and resection margin (R) using the base model

Table S5 C statistic as a measure of model discrimination for various LNR classifications in comparison with the N category for the entire patient collective

Criteria Order	C statistic	C_{SE}	Delta C	P_c
LNR Agnes [13]	0.6364693	0.0208194	0.0152308	0.1194603
LNR Arslan [14]	0.6390056	0.0213279	0.0177670	**0.0421453**
LNR Bagante [15]	0.6218198	0.0219973	0.0005812	0.4713599
LNR Calero [16]	0.6244221	0.0219783	0.0031835	0.3442393
LNR Cao [17]	0.6211064	0.0219581	-0.0001321	0.5072421
LNR Chang [18]	0.6409078	0.0206831	0.0196692	0.0588528
LNR Conci [19]	0.6245013	0.0220587	0.0032628	0.3365385
LNR Fang [20]	0.6340916	0.0210495	0.0128530	0.1419451
LNR Fortea Sanchis [21]	0.6221104	0.0223456	0.0008718	0.4603308
LNR Huang [22]	0.6186891	0.0223129	-0.0025495	0.6157067
LNR Jian Hui [23]	0.6340387	0.0209895	0.0128002	0.1536905
LNR Negi [24]	0.6196270	0.0219352	-0.0016116	0.5801421
LNR La Torre [25]	0.6245938	0.0216094	0.0033553	0.3542798
LNR Lee [26]	0.6366807	0.0207697	0.0154421	0.1053879
LNR Liu [27]	0.6355182	0.0209761	0.0142797	0.1293472
LNR Malleo [28]	0.6258751	0.0215936	0.0046366	0.3110239
LNR Riediger [29]	0.6336689	0.0208742	0.0124303	0.1443088
LNR Rosenberg [30]	0.6239465	0.0215908	0.0027080	0.3833184
LNR Smith [31]	0.6326649	0.0210261	0.0114264	0.1701391
LNR Song [32]	0.6321101	0.0209902	0.0108716	0.1631999
LNR Sun [33]	0.6232464	0.0218289	0.0020079	0.4033919
LNR Wang [34]	0.6273810	0.0211816	0.0061425	0.2792208
LNR Wang [35]	0.6211857	0.0219768	-0.0000528	0.5026851
LNR Xu [36]	0.6294550	0.0213743	0.0082164	0.2154295
LNR Zhou [37]	0.6197987	0.0223085	-0.0014399	0.5667048
N category	0.6212385	0.0218488	0.0000000	NA

NA, not applicable

Table S6 C statistic as a measure of model discrimination for various LODDS classifications in comparison with the N category for the entire patient collective

Criteria Order	C statistic	C_{SE}	Delta C	P_c
LODDS Amini [38]	0.6318195	0.0211029	0.0105810	0.1748382
LODDS Amini [39]	0.6375921	0.0214959	0.0163536	0.0614668
LODDS Bagante [15]	0.6346067	0.0207568	0.0133682	0.1267251
LODDS Calero [16]	0.6404586	0.0215789	0.0192201	**0.0328635**
LODDS Cao [17]	0.6418457	0.0211808	0.0206071	**0.0335283**
LODDS Cao [40]	0.6185702	0.0222217	-0.0026684	0.6212371
LODDS Chang [18]	0.6206969	0.0218971	-0.0005416	0.5213252
LODDS Conci [19]	0.6245674	0.0218755	0.0033288	0.3689825
LODDS Fang [20]	0.6200761	0.0222450	-0.0011625	0.5543410
LODDS Fortea Sanchis [21]	0.6331669	0.0210573	0.0119284	0.1570066
LODDS He [41]	0.6405511	0.0211159	0.0193126	**0.0443455**
LODDS Huang [22]	0.6185041	0.0222723	-0.0027344	0.6232704

LODDS Jian Hui [23]	0.6223878	0.0219051	0.0011492	0.4499498
LODDS Lee [26]	0.6379224	0.0209451	0.0166838	0.0735181
LODDS Persiani [42]	0.6218594	0.0219581	0.0006209	0.4714418
LODDS Ramacciato [43]	0.6168926	0.0222692	-0.0043460	0.6892700
LODDS Riediger [29]	0.6182928	0.0221902	-0.0029458	0.6277033
LODDS Song [32]	0.6388999	0.0210377	0.0176614	0.0656073
LODDS Sun [33]	0.6217141	0.0217800	0.0004755	0.4793164
LODDS Toth [44]	0.6230086	0.0219990	0.0017701	0.4271036
LODDS Wang [45]	0.6249505	0.0214143	0.0037119	0.3649508
LODDS Wang [35]	0.6375789	0.0211583	0.0163404	0.0742148
LODDS Wu [46]	0.6261658	0.0216889	0.0049272	0.3073879
LODDS Xu [36]	0.6200365	0.0222427	-0.0012021	0.5536899
LODDS Xu [47]	0.6380809	0.0211336	0.0168424	0.0609815
LODDS Yang [48]	0.6267206	0.0215700	0.0054820	0.2834110
LODDS Zhou [37]	0.6227709	0.0222931	0.0015323	0.4309522
N category	0.6212385	0.0218488	0.0000000	NA

NA, not applicable
